# Insulin resistance, steatosis and hepatitis C virus

**DOI:** 10.1007/s12072-013-9460-1

**Published:** 2013-08-27

**Authors:** Alessandra Mangia, Maria Ripoli

**Affiliations:** Liver Unit, IRCCS Casa Sollievo della Sofferenza, San Giovanni Rotondo, Italy

**Keywords:** Chronic hepatitis C, Insulin resistance, Type 2 diabetes mellitus, Pegylated interferon, Ribavirin

## Abstract

Epidemiological studies have shown an increased occurrence of metabolic disorders such as insulin resistance (IR) and steatosis in patients with hepatitis C virus (HCV) infection. IR is believed to represent one of the central clinical features of the “metabolic syndrome” and the major pathogenetic factor for type 2 diabetes mellitus. In patients with chronic HCV hepatitis, IR may have several dangerous consequences such as accelerated progression of liver fibrosis, resistance to antiviral therapy and development of hepatocellular carcinoma. According to recent evidence, the global epidemic of metabolic disorders related to incorrect diets will lead physicians to deal with 1.2 billion patients with diabetes in the world in 2025. Given the high prevalence of HCV infection in several countries, metabolic manifestations will contribute to increasing morbidity and mortality in patients with HCV chronic infection in the near future. HCV treatment, shown able to decrease both the occurrence of HCV-related IR and diabetes, may reduce the risk of the associated morbidities.

## Introduction

Hepatitis C virus (HCV) is a positive-strand RNA virus of the Flaviviridae family, responsible for chronic hepatic infection in 160 million people worldwide [1]. Infection may resolve spontaneously in 15–40 % of cases [2], while leading to chronic and progressive disease in the majority of subjects. Cirrhosis and hepatocellular carcinoma (HCC) represent the end-stage liver diseases associated with HCV and are the first indications for liver transplantation in western countries. Although morbidity and mortality associated with HCV are mainly a consequence of HCV-induced liver disease, co-factors associated with HCV infection, including metabolic syndrome, play a relevant additional role.

As shown in the recently published Global Burden diseases risk assessment analysis [3], metabolic syndrome is an increasing phenomenon currently associated with increased mortality. It is reaching pandemic proportions and is expected to increase in particular in East Asia, North Africa and the Middle East, and Latin America. For these reasons, the association between HCV chronic infection and metabolic syndrome may assume dangerous proportions and have devastating consequences in the near future.

## Diabetes

After initial evidence suggesting that the occurrence of type 2 diabetes mellitus (T2DM) in patients with HCV infection was a consequence of impaired glucose metabolism related to cirrhosis [4], large studies have been performed in the general population and in large cohorts of patients followed longitudinally. These studies showed that diabetes is more frequent in patients with HCV infection than in those with HBV, whatever the severity of liver disease [5, 6]. In 2000, in about 10,000 subjects from the NHANES III cohort, representative of the general adult population in the USA, Metha evaluated the prevalence of T2DM in HCV versus non-HCV subjects according to different age classes [5]. The overall prevalence of diabetes in that cohort was 8.2 % and that of HCV 2.1 %. An increase in the prevalence of diabetes was registered with the increase in age with a peak of 30–35 % among HCV-positive subjects aged 60–90. The adjusted odds risk of diabetes in patients with HCV aged 40 or older was 3.77 (95 % CI 1.80–7.8) [5].

The most elegant study on the role of HCV in DM is a further study published by Metha in 2003 [7]. This was a longitudinal study on a cohort of more than 1,000 patients followed for 9 years. The study showed that HCV precedes the development of diabetes in patients with known predisposing conditions such as older age, overweight and male gender. These data support a temporal and causal relationship between hepatitis C infection and diabetes and highlight the promoting role of HCV in patients with other risk factors.

The association between HCV and diabetes with liver disease severity was explored by Hui et al. [8]. In patients with HCV infection and low fibrosis stage (0 or 1), levels of insulin resistance, C peptide and homeostatic model assessment for insulin resistance (HOMA-IR) were significantly higher as compared to matched healthy controls. This concept was further expanded in a recent paper by Maucari et al. [9], who demonstrated the same difference comparing patients with HCV with matched HBV-infected subjects.

A further confirmation of an increased risk of diabetes in patients with HCV infection was derived from studies performed in selected groups of patients such as the recipients of liver or kidney transplant. In these patients, an increased incidence of “de novo” diabetes was registered among HCV-infected as compared to HCV-negative patients [10]. The first report by Knobler in 1998 on liver-transplanted patients demonstrated a four- to eight-fold increase in the occurrence of “de novo” diabetes in HCV-positive patients [11]. Elevated HOMA-IR values and post-transplant diabetes were associated with accelerated progression of fibrosis after transplant [12–14].

According to this evidence, in patients with HCV infection, the risk of insulin resistance and diabetes is doubled per se. A recent meta-analysis combining the results of the massive data on this topic, grouped by study design, confirmed that HCV plays a promoting role in diabetes [15].

Strikingly, Arase et al. [16] recently showed that HCV treatment may decrease the annual incidence of diabetes in HCV-infected patients, independently of other predisposing factors. Once sustained virological response to antiviral treatment has been achieved, T2DM may be prevented. Aghemo et al. [17] confirmed these results, showing that viral eradication after treatment is also able to reduce the occurrence of insulin resistance significantly. Very intriguing results were shown by a recent study demonstrating that danoprevir, an HCV NS3 protease inhibitor, when used as monotherapy, in parallel with a reduction in viral load is able to reduce IR [18]. All these data further support the direct relationship between HCV and glucose metabolism.

## Pathogenesis of HCV-induced insulin resistance

To explain the pathogenesis of HCV-induced insulin resistance, both direct and indirect mechanisms have been investigated. The direct mechanism hypothesizes a direct interference between HCV core proteins and hepatocyte intracellular insulin signaling. As first suggested by Aytug et al. [19] in a landmark study conducted incubating with insulin 42 liver biopsies of non-obese, non-diabetic HCV-positive patients matched with those of 10 non-HCV patients, HCV core protein inhibited the insulin signaling. HCV interferes with serine phosphorylation of insulin receptor-1 (IRS-1) and induces impairment of the downstream tyrosin kinase activity (AKT) signaling pathway and impaired cellular response to insulin. Several studies analyzed the mechanism of HCV-induced IR. In parallel with evidence gathered in vivo, Shintani et al. [20] showed in a transgenic mouse model that insulin fails to suppress hepatic glucose production in animals overexpressing core protein and that IR precedes the occurrence of steatosis.

Recent studies have indicated that the suppressor of the cytokine signaling (SOCS) family that suppresses the insulin signaling cascade through proteosomal degradation and ubiquination of insulin receptor substrate 1 and 2 (IRS) has a key role in IR development in HCV infection [21]. Moreover, Vanni et al. [22] reported an increase in intrahepatic SOCS-3 mRNA expression in subjects with IR. SOCS-3 also inhibits the interferon-induced Janus kinase-signal transducer and suppresses the expression of antiviral proteins, including 2,5-OAS and myxovirus resistance A [23–27]. Increased SOCS-3 expression is reported not only in obese subjects, but also in HCV itself. Finally, the hepatic expression of SOCS-3 predicts the outcome of antiviral therapy, which is significantly increased in non-responder patients as compared with responders [28–31].

At variance with the initial hypothesis that correlated different pathogenetic mechanisms with the different HCV genotypes, currently it seems that SOCS mechanisms are active across all the different genotypes.

More recently, using the euglycemic clamp in combination with the infusion of tracers and indirect calorimetry, it has been shown that an infected liver secretes cytokines or soluble factors able to target distant organs such as muscle and adipose tissue. This evidence supports the existence of peripheral insulin resistance in HCV infection [32].

## HCV, fatty liver and insulin resistance

Hepatic steatosis is another frequent disturbance of lipid metabolism in patients with hepatitis C. Steatosis has been viewed as a characteristic feature of chronic HCV-infected liver, but whether the steatosis is directly related to the presence of HCV or it results from host-related factors remains uncertain. Different pathogenetic mechanisms have been postulated to explain steatosis in patients with HCV infection [33–36]. Moreover, controversial data concerning the relationship between hepatic steatosis and IR exist. Most accredited evidence suggests that HCV itself induces IR development and that steatosis is a consequence of IT. Glucose activates the nuclear transcription factor carabohydrate response. Element-binding protein (ChREBP), by upregulating the conversion of glucose into pyruvate, increases the expression of L-pyruvate. ChREBP increases transcription of the lipogenic enzyme acetyl-CoA carboxylase and fatty acid synthase genes. [37].

In patients with HCV-3, many studies support the hypothesis that steatosis can be directly driven by the virus. Indeed, a direct relationship between HCV replication and steatosis has been observed [38]. To confirm this cause-effect relationship, two concepts have been described: first, the disappearance of steatosis once HCV has been eradicated by treatment [39]; second, the correlation between the severity of steatosis and the level of HCV RNA [39, 40]. Steatosis observed in HCV-3 is believed to be of viral origin, although there are Japanese studies on HCV 1 showing an association between substitution of HCV core amino acid 70 and hepatic steatosis [41, 42].

As shown by Serfaty et al. [43] in mice, impaired lipoprotein secretion may be an independent mechanism explaining lipid accumulation in HCV infection. In this experimental model, HCV core protein interferes with VLDL assembly and secretion by targeting the microsomal triglyceride transfer protein (MTP). MTP plays a limiting role in VLDL assembly, and its inhibition leads to the accumulation of trygliceride alternatively uploaded onto VLDL and secreted. Other studies propose that the fatty acid oxidative pathway is altered. Yasui and others [44, 45] reported that the levels of mRNA and PPARα protein, a nuclear receptor associated with genes implicated in fatty acid oxidative processes, are less expressed in HCV patients with than in those without steatosis. Finally, very recently, the experimental evidence gathered using the euglycemic clamp showed that NEFAs are not increased in patients with HCV as compared to non-HCV infected controls and suggests the existence of different pathogenetic mechanisms [32].

In patients with HCV non-3 genotype infection, fatty liver seems to be related to an increased body mass index [46]. As shown by Fartoux et al. [47], using the HOMA-IR in a cohort of 141 patients, only 28 were infected with genotype 3, and the median HOMA-IR value was significantly higher in HCV-1 patients with steatosis than in those with HCV-3. This evidence supports different mechanisms for steatosis according to HCV genotypes and may also explain the different mechanisms underlying the effects of steatosis on fibrosis. Ultimately, in non HCV-3 genotypes, the effect of steatosis on liver fibrosis seems to depend on the pathogenesis of fat accumulation.

Recent findings on the role of genetic variants associated with different responses to interferon-based treatment, the single-nucleotide polymorphisms near the IL28B gene, shed further light on the pathogenetic mechanism of steatosis in HCV infection, suggesting that different genetic backgrounds may be conditions for the development of steatosis regardless of the genotype in HCV-infected subjects. Indeed, in liver biopsies of patients with HCV 1 infection, it has been shown that steatosis is less represented in carriers of the IL28B CC genotype, which predicts a favorable response to interferon therapy [48]. This evidence confirms previous data demonstrating a reduction in serum trygliceride levels and an increase in serum LDL cholesterol levels in patients with IL28B CC [49]. Moreover, an association between CC and lower gamma-GTP levels has also been shown. Because gamma-GTP tends to be elevated in patients with steatosis, these results suggest that the lower levels of this liver enzyme may be considered a consequence of a lower degree of steatosis associated with CC. The lower levels of lipids in these patients seem to be due to a more efficient export of lipids from cells in carriers of IL28B CC [50].

## Clinical consequences of IR

### Fibrosis severity and progression

According to the reported evidence, the interplay between HCV and metabolic disorders induces liver disease complications; first of all, it accelerates the progression of fibrosis. A close relationship between IR and liver fibrosis has been shown in many studies. Hui et al. [8] reported that, in 260 patients with chronic hepatitis C, the HOMA-IR score, but not steatosis, was associated with the severity of fibrosis and accelerated the rate of progression. Therefore, regardless of the viral genotype and severity of liver damage, serum insulin concentrations and HOMA-IR index scores increase with the severity of hepatic fibrosis. Petta et al. found that in HCV genotype 1 patients, HOMA-IR scores >2.7 and platelet levels <200 × 10^3^/μl were the diagnostic criteria for severe fibrosis (F3 and F4) [51]. The association between IR and hepatic fibrosis exists independently of the exclusion of cirrhotic patients from the analysis or of the adjustment for other factors associated with fibrosis, including steatosis [52]. This suggests that insulin is a driving force behind accelerated fibrosis progression in patients chronically infected with HCV [53]. Hyperinsulinemia and hyperglycemia directly stimulate hepatic stellate cells, leading to activation of connective tissue growth factor and subsequent accumulation of extracellular matrix [54, 55]. In addition, leptin and TNF-α could be the molecules responsible for accelerated fibrosis. Patients diagnosed with IR frequently show hyperleptinemia and increased serum TNF-α levels; both of these are also able to activate hepatic stellate cells, leading to increased fibrogenesis [56, 57].

The hypothesis of an association between steatosis and fibrosis progression in HCV-infected patients needs to be confirmed. Indeed, while several studies claimed that there is an association between steatosis and fibrosis progression, very few evaluated the association with a proper methodological approach. Thus, only longitudinal studies investigating paired biopsies might guarantee sound results on this association. Of interest, in the Maid study, a large multicenter cohort study involving HCV patients infected with multiple genotypes from different countries around the world, it was demonstrated that while the main predictor of fibrosis in patients with genotype 1 is the presence of diabetes, in HCV-3 a higher grade of inflammatory activity is independently associated with fibrosis progression [58].

### HCC development

In patients with diabetes, HCV is an independent factor for the development of HCC, even after adjusting for the confounding variables of age and gender [59, 60]. It is well recognized that in males a HOMA-IR score >3 is an independent predictor of HCC [61, 62]. The molecular mechanisms underlying the link between IR and HCC remain unclear and include the mitogenic role of insulin and its stimulating property on cell proliferation [63]. Additional hypotheses are based on the binding of insulin with insulin-like growth factor (IGF)-1 receptor, resulting in the activation of tyrosine kinase and a cascade of downstream intracellular responses [63]. The question is now whether the coexistence of HCV increases the risk of cancer in patients with metabolic syndrome. Strikingly, insulin resistance and obesity are associated with a higher risk of adenocarcinoma, including colon cancer [63]. The long duration of chronic HCV diseases hampers designing effective studies. Therefore, it is not surprising that a number of small studies evaluating the association among HCV, diabetes and HCC reached contrasting results on this debated aspect. In paired biopsies of patients with HCV who did or did not develop cancer, Kumar et al. [64] failed to show an association with diabetes. In contrast, a study performed by Pekow [65] on explanted livers of patients with cancer demonstrated an association between a more severe liver steatosis and the presence of cancer. A promoting role of HCV has been shown in a large European multicenter study conducted by Veldt et al. [66]. In this study, the coexistence of diabetes increased the frequency of liver cancer in comparison to HCV infection alone [66]. In contrast, in a study on 82,000 veterans with HCV infection and a comparable number of non-HCV-infected patients, despite a lower frequency of hypercholesterolemia, hypertrygliceridemia, hyperlypemia and hypertension in HCV-infected patients, a significant increase in the risk of liver cancer in the presence of a higher degree of steatosis was demonstrated [59].

### Atherosclerosis development

A further question on the interactions between HCV and metabolic disorders is related to the possible increased risk of cardiovascular morbidity and mortality in patients with chronic hepatitis C. The results so far accumulated on this topic are contradictory. In a large European survey, the risk of arterial damage observed in patients with HBV was comparable to that of patients with HCV infection [67]. However, recently, exploring surrogate markers of cardiovascular mortality, evidence of intima-media thickness (IMT) and carotid plaques was investigated [68]. In the Reveal HCV cohort, a 1.5 risk of cardiovascular-related deaths was registered in Taiwan [69]. The association was significantly higher in patients with active HCV RNA replication [64]. Petta et al. [70] evaluated the prevalence of carotid atherosclerosis compared with a control population in order to assess the potential association among atherosclerosis, host and viral factors, and liver histological features. Out of 174 consecutive G1 CHC patients, 73 (41.9 %) showed carotid plaques at a rate significantly higher than that of control subjects (22.9 %) (*p* < 0.001). Similarly, HCV-1 chronic hepatitis C patients had a greater IMT compared with control patients (1.04 ± 0.21 vs. 0.90 ± 0.16; *p* < 0.001). Multivariate logistic regression analysis showed that older age [odds ratio (OR) 1.047, 95 % confidence interval (CI): 1.014–1.082, *p* = 0.005] and severe hepatic fibrosis (OR 2.177, 95 % CI:1.043–4.542, *p* = 0.03) were independently linked to the presence of carotid plaques.

### Reduced response to interferon treatment

The treatment of metabolic disorders in HCV-infected patients coincides with the treatment of HCV. However, among the clinical consequences of HCV-induced IR, there is an impaired response to antiviral treatment [71]. Although not unanimously accepted, rates of response to antiviral treatment, including pegylated interferon and ribavirin, are inversely associated with HOMA-IR index scores [72]. An increased HOMA-IR index score is associated with reductions in 24-h virological response, early virological response (EVR) and sustained virological response (SVR) to antiviral therapy [73]. The relevance of IR in antiviral therapy based on the combination of pegylated interferon and ribavirin has been reported, regardless of the HCV genotype [74, 75].

An increase of HCV RNA levels and reduced rates of SVR, irrespectively of other response predictors, have indeed been registered in HCV-infected patients.

Several studies evaluated the negative effect of IR on SVR. The first evidence was reported by Romero-Gomez et al. [76] in patients with HCV-1 infection. A French meta-analysis recently showed that the rate of SVR is significantly reduced by the severity of IR in patients with HCV infection overall and in patients with genotype 1 in particular [77]. However, the study showed an inverse correlation between the severity of IR and SVR in both HCV patients overall and patients with HCV-1 in particular [77]. The impact of HOMA-IR in patients with genotype 2 and 3, in case of dual treatment with pegylated interferon and ribavirin, was recently explored by Poustchi et al. [78]. In these patients, a six-fold risk of non-response in case of a HOMA-IR value higher than 2 was observed.

Many authors have been trying to increase insulin sensitivity with the aim of increasing SVR. In particular, antidiabetic drugs such as metformin and thiazolidinediones have been used to improve insulin sensitivity. In a multicenter study, treatment non-responders were re-treated with pegylated interferon-α, ribavirin and pioglitazone. An early termination of this study was required as none of the patients showed a virological response at week 12 despite the improvement in insulin sensitivity [79]. Another analysis in naïve non-diabetic patients who received a lead-in regimen of pioglitazone monotherapy for 4 weeks, followed by pioglitazone added to standard therapy with pegylated interferon-α and ribavirin, revealed that, despite an increase in rapid virologic response, no significant effect on SVR can be appreciated. [80]. Treatment with metformin or thiazolidinediones, in addition to antiviral therapy, will probably be less important in the future, when more potent antivirals, such as the combination of different direct inhibitors of HCV enzymes, will be used in clinical practice. Currently, the uselessness of insulin sensitizers to reduce the severity of IR before starting an antiviral treatment plays against the idea that insulin resistance per se reduces SVR.

According to the knowledge accumulated so far, the only way to obtain an increase in the rates of SVR in these patients may be to advise weight loss before starting antiviral treatment [81]. As shown in patients without concomitant viral hepatic infections, only exercise ensures both fat deposit and liver enzyme decreases.

IR can be influenced by lifestyle changes. It is known that weight loss can improve different clinical features of metabolic syndrome, including insulin sensitivity, irrespectively of the pathogenesis. Moreover, a study on 19 subjects showed that weight loss may also improve fibrosis in CHC patients [82]. Aerobic exercise improves HOMA-IR values and decreases body fat in patients with chronic hepatitis C.

As anticipated, with the use of triple combination treatment including protease inhibitors, the role of IR showed a minor impact. Strikingly, danoprevir response is not reduced by insulin resistance status [83]. As demonstrated by Serfaty et al. [84] in naïve HCV-1 patients receiving triple treatment with pegylated interferon, ribavirin and telaprevir, HOMA-IR values were not predictive of virological response, while sustained virological response appeared to be associated with improved HOMA-IR scores. These results suggest that metabolic factors and insulin resistance do not have a significant effect on telaprevir-based treatment efficacy.

These data led us to expect that a pre-treatment evaluation of HOMA-IR, encouraged by the previously reported meta-analysis results, may be less relevant in the future workup of patients undergoing triple treatment regimens.

In our experience, higher body mass index and severe liver damage have been associated with higher rates of relapse in patients with HCV-2 and 3 receiving short treatments [85]. Therefore, lifestyle changes are urgently recommended in patients such as those with genotype 2 dealing with dual treatment in the immediate future. The forthcoming analyses of data obtained in the Fission study with all oral interferon-free regimens combining sofosbuvir with ribavirin in patients infected with HCV-2 and 3 may shed further light on the future role of both HOMA-IR and dieting in patients infected with these genotypes.

## Conclusions

Hepatitis C is associated with IR, which in turn contributes to the progression of fibrosis, the development of HCC and perhaps an increased risk of cardiovascular diseases. IR may lead to the development of diabetes in subjects with HCV infection and predisposing factors.

Treatment of HCV may reduce the burden of metabolic disorders. In patients infected with HCV-1, the use of more efficient therapies than the combination of pegylated interferon and ribavirin will decrease the need for a pre-treatment HOMA-IR assessment in order to define a correct prediction of SVR. In contrast, in patients with HCV-2 and 3 still receiving the combination of pegylated interferon and ribavirin as standard of care treatment, a correct pre-treatment assessment of the risk of diabetes together with advice on exercising to reduce body weight might reduce the risk of relapse.
